# Gremlin-1 Overexpression in Mouse Lung Reduces Silica-Induced Lymphocyte Recruitment – A Link to Idiopathic Pulmonary Fibrosis through Negative Correlation with CXCL10 Chemokine

**DOI:** 10.1371/journal.pone.0159010

**Published:** 2016-07-18

**Authors:** Katri Koli, Eva Sutinen, Mikko Rönty, Pia Rantakari, Vittorio Fortino, Ville Pulkkinen, Dario Greco, Petra Sipilä, Marjukka Myllärniemi

**Affiliations:** 1 Research Programs Unit, Translational Cancer Biology, University of Helsinki, Helsinki, Finland; 2 Transplantation Laboratory, Haartman Institute, University of Helsinki, Helsinki, Finland; 3 University of Helsinki and Helsinki University Hospital, Heart and Lung Center, Department of Pulmonary Medicine, Helsinki, Finland; 4 Department of Pathology, University of Helsinki and Fimlab laboratories, Pathology, Tampere, Finland; 5 MediCity Research Laboratory, University of Turku, Turku, Finland; 6 Unit of Systems Toxicology and Nanosafety Centre, Finnish Institute of Occupational Health (FIOH), Helsinki, Finland; 7 Department of Physiology, Institute of Biomedicine and Turku Center for Disease Modeling, University of Turku, Turku, Finland; French National Centre for Scientific Research, FRANCE

## Abstract

Idiopathic pulmonary fibrosis (IPF) is characterized by activation and injury of epithelial cells, the accumulation of connective tissue and changes in the inflammatory microenvironment. The bone morphogenetic protein (BMP) inhibitor protein gremlin-1 is associated with the progression of fibrosis both in human and mouse lung. We generated a transgenic mouse model expressing gremlin-1 in type II lung epithelial cells using the surfactant protein C (SPC) promoter and the Cre-LoxP system. Gremlin-1 protein expression was detected specifically in the lung after birth and did not result in any signs of respiratory insufficiency. Exposure to silicon dioxide resulted in reduced amounts of lymphocyte aggregates in transgenic lungs while no alteration in the fibrotic response was observed. Microarray gene expression profiling and analyses of bronchoalveolar lavage fluid cytokines indicated a reduced lymphocytic response and a downregulation of interferon-induced gene program. Consistent with reduced Th1 response, there was a downregulation of the mRNA and protein expression of the anti-fibrotic chemokine CXCL10, which has been linked to IPF. In human IPF patient samples we also established a strong negative correlation in the mRNA expression levels of gremlin-1 and CXCL10. Our results suggest that in addition to regulation of epithelial-mesenchymal crosstalk during tissue injury, gremlin-1 modulates inflammatory cell recruitment and anti-fibrotic chemokine production in the lung.

## Introduction

Gremlin-1, also known as Drm, is a gene involved in kidney and lung branching morphogenesis and in bone development [1]. It is a glycosylated cysteine knot protein and belongs to the DAN family of bone morphogenetic protein (BMP) inhibitors [2]. Gremlin-1 binds with high affinity to BMP-2 and -4 and with lesser affinity to BMP-7 [3]. This binding blocks receptor association and signaling by the BMP isoforms. During development inhibition of BMP-4 signaling by gremlin-1 is crucial for the proximal-distal patterning in the lung. Gremlin-1 knockout mice die in utero due to the lack of kidneys and lung septation defects [1]. Overexpression of gremlin in the distal lung epithelium under the surfactant protein C (SP-C) promoter leads to proximalization of distal lung tubules [4]. These studies suggest that gremlin-1 is crucial for the epithelial-mesenchymal feedback signaling during lung development.

Gremlin-1 expression has been associated with many diseases, which are characterized by reactivation of embryonic programs. In normal adult lung gremlin-1 expression is low [5]. We have shown high gremlin levels in the lungs of idiopathic pulmonary fibrosis (IPF) patients and this correlates with poor pulmonary function tests [5, 6]. IPF is an aggressive form of pulmonary fibrosis characterized by scar formation, activation of alveolar epithelial cells, accumulation of fibroblasts and extracellular matrix leading to loss of lung function. Rescue of BMP signaling by administration of BMP-7 or tilorone reduces significantly fibrosis in an experimental silica-induced fibrosis in mice [7, 8]. Furthermore, Farkas et al. [9] have shown that transient overexpression of gremlin-1 in rat lung results in epithelial activation and the appearance of fibroblastic foci, highlighting the role of gremlin-1 in fibrosis development.

Gremlin-1 and aberrant BMP signaling has been functionally linked to fibrosis also in the kidney, heart and liver [10–12] as well as fibrotic complication in the eye [13]. It also plays an important role in pulmonary hypertension [14]. In addition, recent studies also indicate an upregulation of gremlin-1 in epithelial cancers including lung carcinomas [15, 16]. We have shown that gremlin-1 is involved in the regulation of cell plasticity and chemoresistance in mesothelioma [17]. Inhibition of BMP-mediated signaling plays an important role during development and disease progression. By blocking the differentiation inducing BMP signals gremlin-1 allows proliferation and maintains stem cell properties [18]. On the other hand, gremlin-1 induces an epithelial-to-mesenchymal transition (EMT) phenotype in cells, which is involved in fibrotic processes and cancer cell migration, invasion and chemoresistance [5, 19]. This is likely mediated partly by BMP-independent functions of gremlin-1. Gremlin-1 is also a proangiogenic factor stimulating endothelial cells in a BMP-independent manner [20]. Both pro- and anti-inflammatory functions have been described for gremlin-1. It can inhibit monocyte migration by interacting with Slit proteins [21] and block macrophage differentiation by interacting with macrophage inhibitory protein (MIF) [22, 23]. Pro-inflammatory response has been described in endothelial cells through VEGFR2 activation [24] indicating that modulation of inflammation associated processes is highly context dependent.

Here, we produced a transgenic mouse with type II epithelial cell specific overexpression of gremlin-1 to study adult lung homeostasis and injury responses. Surprisingly, gremlin-1 did not induce fibrosis or potentiate particulate-induced fibrosis. Gremlin-1 is shown to regulate inflammatory interferon responses and anti-fibrotic chemokine production in response to particulate exposure, which is a new pro-fibrotic mechanism of action for gremlin-1.

## Materials and Methods

### Antibodies

The antibody against mouse gremlin was from R&D Systems (Minneapolis, MN; AF956). Antibodies used for the detection of lymphocytes were from BD Biosciences (Franklin Lakes, NJ; anti-CD4, clone H129.19; anti-CD8, clone 53–67) and eBioscience (San Diego, CA; anti-CD45R, clone RA3-6B2). CD11b antibody was from Abcam (Cambridge, UK; EPR1344) and myeloperoxidase antibody form Santa Cruz (M-17).

### Construction of SPC+loxP-PGK-tn5-NEO-pA-loxP-mGremlin1-pA plasmid

Gremlin-1 cDNA was cloned under the 3.7-kb hSPC-promoter (kindly provided by Jeffrey Whitsett, Perinatal Institute, Cincinnati, OH [25]). A loxP-PGK-tn5-NEO-Stop-loxP cassette (GeneBridges, Heidelberg, Germany) was cloned between gremlin-1 cDNA and hSPC promoter with Red/ET recombineering [26].

### Transgenic mice and tissue preparation

All experiments involving animals were approved by the Provincial State Office of Southern Finland (ESAVI/871/04.10.03/2012) and carried out in accordance with institutional guidelines, which fulfill the requirements defined in regulations of the Finnish Act on the Protection of Animals used for Scientific or Educational Purposes (497/2013) and were performed according to the 3R. Age and sex-matched animals were used in all experiments.

The SPC-lox-gremlin1 transgenic mouse strain was produced by pronuclear injection in a C57BL/6N background at the Turku Center for Disease Modeling (University of Turku, Finland). From 5 transgenic lines number #105 was estimated by quantitative PCR analyses to have the highest transgene copy number (4–5) and was selected for further characterization. This SPC-lox-gremlin1 strain has been archived in the European Mouse Mutant Archive EMMA (EM:08364). The mice were crossbread with R26CreERT strain [27] and the litters genotyped by using Phire Animal Tissue Direct PCR Kit (Thermo Fisher Scientific, Waltham, MA) and the following primers (Oligomer, Helsinki, Finland): For 5’-TTC CCT GCC ACA GTC TGA GAG C-3’ and Rev 5’ GGC GGA TTT GTC CTA CTC AGG AGA GCG-3’ for SPC-lox-gremlin1 as well as For 5’-GCA CGT TCA CCG GCA TGA AC-3’ and Rev 5’-CGA TGC AAC GAG TGA TGA GGT-3’ for R26CreERT. During the initial characterization double positive mice were treated with tamoxifen (Sigma-Aldrich, St. Louis, MO; 50 mg/kg/day ip., five consecutive days) to induce Cre-mediated recombination. Deletion of the floxed NEO cassette from SPC-lox-gremlin1 was monitored using the following primers: For 5’-GGC TTG GTC CTT CAC CTC TGT-3’ and Rev 5’-GTG TGA CCA TCA TGG TGG TGA AC-3’. Tamoxifen treatment was omitted from further experiments due to the activation of gremlin expression also without tamoxifen. The mice were euthanized using carbon dioxide. The left lung was split into two symmetrical pieces, where one piece was fixed in 4% PFA, and the other processed for frozen sections.

### *In vivo* silica exposure and histological scoring

Wild type and transgenic mice were exposed to silicon dioxide (silica) (Sigma, St. Louis, MO, 2.5 mg/50 μl sterile PBS). Two subsequent oropharyngeal aspiration doses were given on days 0 and 3 of the experiment to ensure even exposure of all animals. In the first experiment 4 months old mice were followed up after 2 months of silica-exposure and sacrificed (n = 5 in each group). In an independent, shorter experiment the mice were exposed to silica from 4 weeks to 6 weeks of age (n = 8 in each group). At sacrifice, the entire left lung was collected for histological analysis (fixed in 4% PFA and embedded in paraffin for sectioning). The entire right lung was frozen in dry ice and later homogenized for further analysis.

When present, the total number of lymphocytic foci were calculated from each lung section. The remaining inflammatory cells (mainly neutrophils and occasional eosinophils) were calculated semi-quantitatively. Grading was from 0–4: 0. no inflammation, 1. few scattered inflammatory cells, 2. few inflammatory cell foci and scattered inflammatory cells, 3. many inflammatory cell foci throughout the lung, and 4. severe inflammation throughout the lung tissue. Fibrosis was scored with a similar grading from 0–4: 0. no fibrosis, 1. few small fibrotic foci without interruption of the lung architecture, 2. moderate fibrosis with several larger fibrotic foci, 3. heavy fibrosis with large fibrotic patches and disruption of normal lung architecture, and 4. intense fibrosis with significant derangement of the lung parenchyma. Scoring of fibrosis was performed by two observers who scored blindly and independently all samples. Pleural thickening was quantitated separately using a similar scoring system from 0–4. 2–3 sections / mouse left lung were analyzed. All animals from all different groups were analyzed, and the mean value of each scoring was used for statistical analysis.

### Collection and analyses of bronchoalveolar lavage (BAL) fluid

Bronchoalveolar fluid lavage was performed to euthanized mice by cannulating the trachea and washing with 2x300 μl PBS. Differential cell counts were obtained by microscopy of May-Grünwald-Giemsa-stained cytocentrifuge preparates: 150 μl BALF was loaded to cytospin chambers containing Superfrost Ultra Plus glass slides (Menzel GmbH & Co KG, Braunschweig, Germany) and centrifuged for 8 minutes, 500 rpm.

Equal volumes of BALF from three silica-treated wild type or transgenic mice were pooled and used to analyze differences in cytokine levels. Commercial Mouse Cytokine Array Panel A was from R&D Systems and used according to the manufacturer’s instructions. Quantity One version 4.6 (BioRad, Hercules, CA) was used for quantification. The results are expressed as averages of two replicates.

### Immunohistochemistry and immunofluorescence

Paraffin-embedded tissue samples were processed and stained using the Novolink Polymer Detection System (Novocastra, Leica Biosystems, Newcastle upon Tyne, UK) and visualized by diaminobenzidine (DAB, Vector Laboratories) as described previously [28]. For CD11b, CD45R and myeloperoxidase staining, the formalin-fixed paraffin embedded specimens were stained on Leica BOND-MAX fully automated staining system as defined in the manufacturer’s staining protocol [with the Leica Bond Polymer Refine Detection-kit, Bond Epitope Retrievel Solution 2, 20 mins, R.T.U. Normal Horse serum 2,5% (Vector Laboratories, Burlingame, CA) blocking] or manually by using Vectastain Elite ABC Kit (RatIgG) (Vector Laboratories, Burlingame, CA) and ImmPRESS (Anti-Goat IgG) Kit (Vector Laboratories, Burlingame, CA). Images of IHC sections were captured with Nikon DS-Fi1 (Nikon, Amsterdam, Netherlands).

Immunofluorescence staining of frozen lung tissue sections was done using gremlin-1, anti-CD4 or anti-CD8a primary antibodies. Visualization was performed with Alexa Fluor 488 or Alexa Fluor 568 conjugated secondary antibodies (Invitrogen, Thermo Fisher Scientific, Waltham, MA USA) followed by mounting with a Vectashield Hardset Mounting medium with DAPI (Vector Laboratories, Burlingame, CA). Gremlin-1 staining was performed from lung tissue of all animals included in experiments to ensure that the transgene was expressed as expected.

### Western blotting

Pulverized lung tissue was lysed in RIPA buffer containing Pierce protease inhibitor cocktail (Thermo Scientific, Rockford, IL). Equal amounts of protein were separated by SDS-PAGE using 4–20% gradient Tris-HCl polyacrylamide gels (Bio-Rad). Electrophoretically separated proteins were transferred to nitrocellulose membranes using semi-dry blotting system (Bio-Rad). Immunodetection was carried out as described [7].

### RNA isolation and quantitative RT-PCR

Total RNA from tissue or cells was isolated using RNeasy Mini kit (Qiagen, Hilden, Germany) and reverse transcribed to cDNA using iScript cDNA synthesis Kit (Bio-Rad, Hercules, CA) according to the manufacturer's instructions. The cDNAs were amplified using TaqMan Assays-on-Demand gene expression products (Applied Biosystems, Waltham, MA) and CFX96 Real-time PCR detection system (Bio-Rad). The relative gene expression differences were calculated with the comparative delta delta cycle threshold (ΔΔCT) method and the results have been expressed as mRNA expression levels normalized to the levels of a gene with a constant expression (TBP, TATA-binding protein). The results are expressed as box plots, where the middle bar represents median and the upper and lower boundaries of the box represent the 25^th^ and 75^th^ percentile of the values. The whiskers in box plots represent minimum and maximum values.

### Transcriptional profiling and data analysis

Total RNA from lung tissue was isolated using RNeasy Mini kit (Qiagen). RNA integrity was confirmed using an Agilent 2100 Bioanalyzer (Agilent Technologies, Santa Clara, CA). Gene expression analysis (n = 4 in each group) was performed using Agilent SurePrint G3 Mouse Gene Expression 8x60K microarrays according to the manufacturer’s instructions at the Biomedicum Functional Genomic Unit (Helsinki, Finland). The microarray data have been deposited in NCBI Gene Expression Omnibus (GEO) database [29] and are accessible through GEO Series accession number GSE80406. Raw data was quality checked according to the Agilent standard procedures. The median foreground intensities were imported into the R software version 3.0.0 (http://cran.r-project.org) [30] and analyzed with the BioConductor package limma [31]. Log2 transformation and quantile normalization was performed on the single channel data separately, according to the suggestions by Smyth and Altman [32]. Background correction was not carried out, as suggested by Zahurak et al. [33]. Differentially expressed genes were identified by using linear models and empirical Bayes pairwise comparisons [34].

The functional categorization of DE genes was performed using a novel R-based package namely BACA [35]. It queries the DAVID knowledgebase and build a charts showing multiple enrichment analysis results across different conditions/treatments. Each annotation in the chart is represented as a circle (or bubble) that has a size, indicating how many genes in a list of DE genes are associated with it, and a color indicating whether the genes are down- (default color is green) or up- (default color is red) regulated.

### Human tissue samples

Written informed consent from patients and an approval for collecting clinical samples was received from the Helsinki University Hospital Ethics Board (HUS 426/13/03/01/09). The study was conducted according to the principles outlined in the Declaration of Helsinki. A permission to use tissue samples from deceased patients was obtained from the national supervisory authority of welfare and health (3317/05.01.00.06/2011). Patient characteristics and details have been published in [36].

### Cell culture

CCL-190 normal lung fibroblasts and CCL-191 and CCL-134 IPF fibroblasts were obtained directly from ATCC (Manassas, VA). IPF fibroblasts named UIP-IV fibroblasts were isolated as previously described [5]. All cells were cultured in Dulbecco’s Modified Eagles’s Medium (Sigma) supplemented with 10% fetal bovine serum (Gibco, Paisley, UK) and antibiotics (Gibco).

### Statistical analysis

All comparisons were made using nonparametric tests with SPSS version 23 software (IBM). Multiple group comparisons were made using Kruskal-Wallis test, and two-group comparisons were made using Mann-Whitney U-test. Correlation coefficients (Spearman) were calculated using SPSS. P values below 0.05 were considered statistically significant.

## Results

### Transgenic expression of gremlin-1 in mouse lung

**Fig 1:**
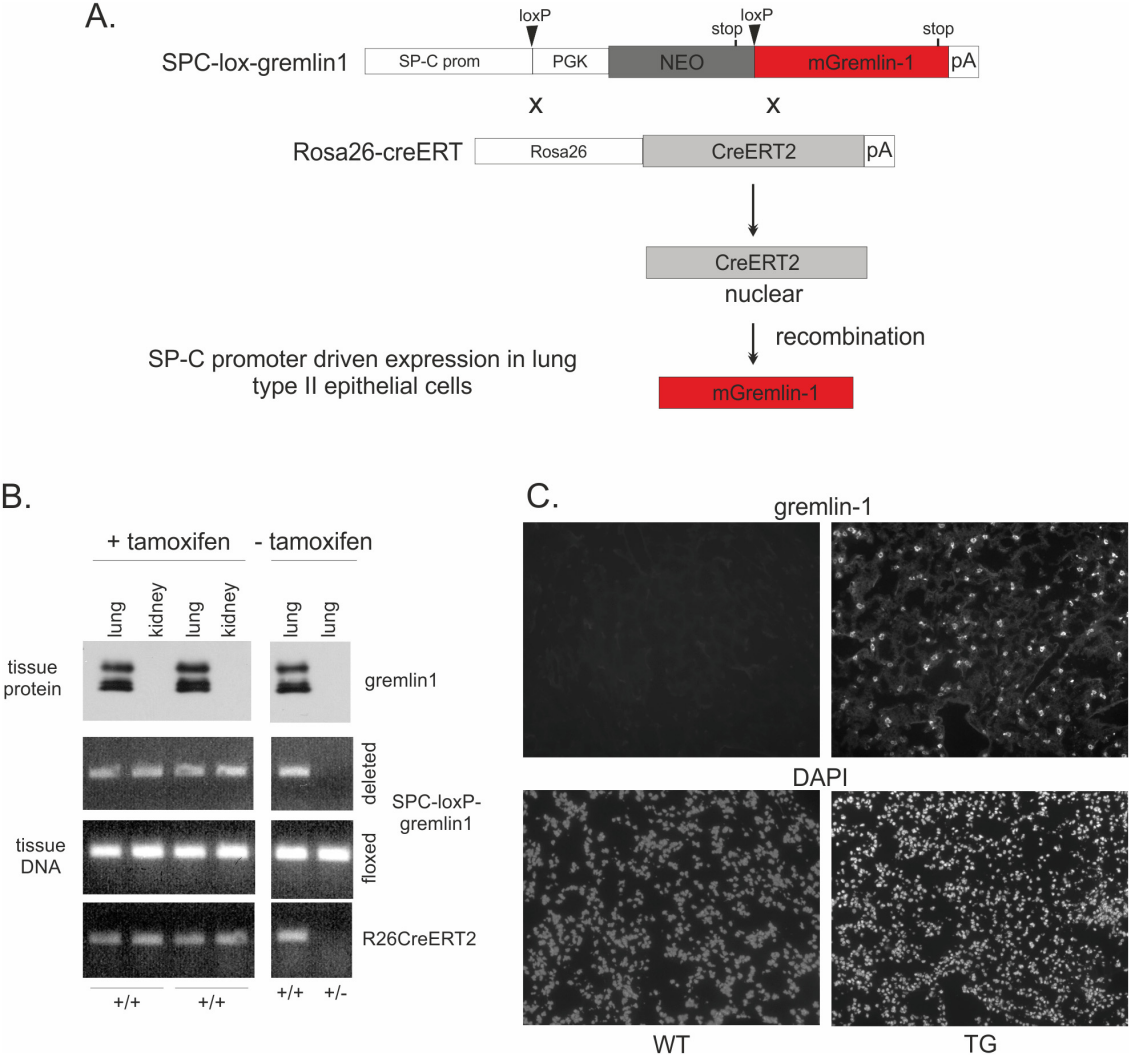
Generation of gremlin-1 transgenic mice. A. Schematic representation of the breeding strategy. B. SPC-lox-gremlin-1 mice were crossbread with Rosa26CreERT mice. Part of the mice were treated with tamoxifen for five days before they were sacrificed. Tissue DNA was isolated followed by genotyping for SPC-loxP-gremlin1 (floxed) and R26CreERT2. The recombination event was monitored using primers surrounding the NEO cassette (deleted). Results of SPC-lox-gremlin-1/ R26CreERT2 positive (+/+) and SPC-lox-gremlin-1/- positive (+/-) mice are shown. Expression of gremlin-1 protein was analyzed using Western blotting of lung and kidney tissue lysates. C. Gremlin-1 protein expression was analyzed by immunofluorescence staining of frozen lung tissue sections. Original magnification 200x. WT = wild type mice; TG = gremlin-1 transgenic mice.

To study the effects of gremlin-1 overexpression on adult lung homeostasis and injury repair, a transgenic mouse expressing gremlin-1 under the surfactant protein C (SPC)-promoter was generated. Since gremlin-1 expression is critical for lung development [1], we used the *Cre-LoxP* system for the activation of transgenic gremlin-1 expression in adult lung (Fig 1A, see Methods). SPC-lox-gremlin1 mouse was crossed with the Rosa26-CreERT2 mouse expressing the Cre recombinase fused to mutant estrogen receptor [27]. Mice positive for both transgenes are from hereon called gremlin-1 transgenic mice. Western blotting of tissue lysates indicated that gremlin-1 was abundantly expressed in transgenic lungs but not in the kidneys suggesting specific targeting of protein expression to the lung by the SPC-promoter (Fig 1B). Gremlin-1 expression was not activated in SPC-lox-gremlin1 mice without the Cre transgene (Fig 1B). To our surprise, tamoxifen treatment was not needed to activate the transgene expression. This suggests that some of the robustly expressed CreERT2 fusion protein probably enters the nucleus and induces the recombination event even in the absence of tamoxifen. Gremlin-1 localization was then studied by immunofluorescence staining of lung tissue. In wild type mice gremlin-1 was not detectable. In transgenic mice the staining pattern was consistent with alveolar type II cell localization of gremlin-1 in 6 week old mice (Fig 1C). The transgene expression was activated after birth. At E17 no gremlin-1 staining was observed, whereas at P0 low intensity staining was detected. Thereafter gremlin-1 staining was clearly seen in transgenic lungs (S1 Fig).

**Fig 2:**
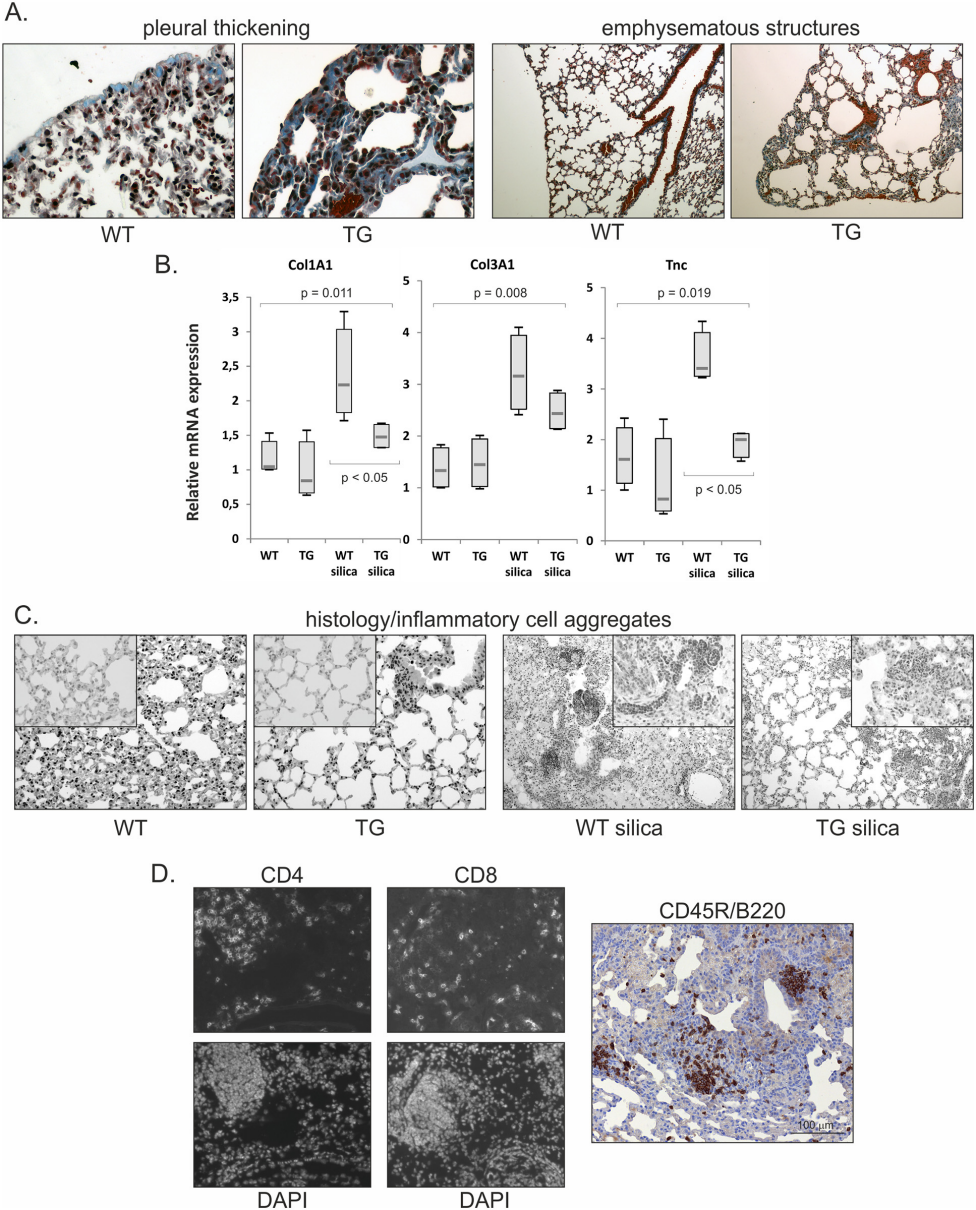
Reduced amount of inflammatory cell aggregates in gremlin-1 transgenic lung. A. Histological sections of gremlin-1 transgenic and wild type lung showing pleural thickening (original magnification 400x) and alveolar space enlargement (original magnification 100x) at 6 months of age. Results of histological scoring are presented in Table 1. B. Mice were treated with silica for 2 months and sacrificed at 6 months of age. Collagen 1 (*Col1A1*), collagen 3 (*Col3A1*) and tenascin-C (*Tnc*) mRNA expression levels analyzed from lung tissue RNA using quantitative RT-PCR (n = 4). The results are presented as box blots. The p values were calculated using the Kruskal-Wallis test or the Mann-Whitney U-test when comparing two groups. WT = wild type mice; TG = gremlin-1 transgenic mice. C. Representative histological pictures showing decreased amount of inflammatory cell aggregates in silica-treated gremlin-1 transgenic mice. Original magnification 200x, inset original magnification 400x. D. Immunofluorescence staining of wild type frozen lung tissue sections using CD4 and CD8 T-cell markers. DAPI staining was used to visualize the nuclei. Immunohistochemical staining of CD45R/B220 (brown color) is shown on the right.

Gremlin-1 transgenic mice were viable and the phenotypic changes observed were very mild. Mice did not show differences in body weight, signs of respiratory insufficiency or any notable alterations in well-being (data not shown). Histological staining of lung tissue indicated slight pleural thickening and possible alveolar space enlargement at 6 month old animals (Fig 2A and Table 1). Since gremlin-1 was expressed soon after birth, it is possible that these alterations were caused by interference with postnatal lung development. Occasionally, in some of the one year old transgenic animals we observed aberrantly localized arterial structures in the peripheral lung.

**Table 1:** Histological scoring.

	Fibrosis /score	Emphysematous structures/score	Pleural thickening/score	Inflammatory cells/aggregates per section
**WT**	0	0,63±0,13	**0,50±0,29**	0,5±0,29
**TG**	0,13±0,13	1,38±0,52	**1,63±0,38**^a^	0,25±0,25
**WT+silica**	2,38±0,24	**0,25±0,25**	**0,75±0,32**	**12,75±4,84**
**TG+silica**	2,00±0,32	**1,50±0,35**^b^	**1,30±0,12**^a^	**4,0±3,03**^b^

^a^ p = 0.06 compared to WT or WT + silica;

^b^ p < 0.05 compared to WT + silica

### Reduced lymphocyte recruitment after silica-exposure in gremlin-1 transgenic mice

As we have previously shown that gremlin-1 overexpression is related to human IPF progression, we studied the response of gremlin-1 transgenic mice to exposure to silicon dioxide (silica), a fibrosis-inducing particulate matter. Oropharyngeal aspiration of silica induces a neutrophilic inflammatory response which is followed by progressive lung fibrosis without affecting animal mortality or well-being [36, 37]. Transgenic and wild type mice were exposed to silica at the age of 4 months, sacrificed after two months and analyzed for alterations in inflammatory and fibrotic responses. Gremlin-1 overexpression was not associated with an increased fibrotic response at two months after exposure. Histological fibrosis score was not different between silica-exposed transgenic and wild type mice (Table 1). Interestingly, there was reduced expression of some extracellular matrix genes (*Col1A1* and *Tnc*) indicating subtle changes in the fibrotic response (Fig 2B). *Tnc* showed a trend towards decreased expression also in non-exposed transgenic mice. In 8 week old transgenic mice a similar, statistically significant, decrease was noted (S2A Fig).

Neutrophils were stained from lung tissue sections using myeloperoxidase as a marker. Silica-treated transgenic lungs showed decreased myeloperoxidase staining score (1.87 ± 0.31 SEM), but the difference to wild type lungs (2.69 ± 0.08 SEM) was not statistically significant. Scoring of inflammatory cell aggregates in lung tissue sections indicated a reduced number of mononuclear cell aggregates in transgenic mice (Table 1, Fig 2C), indicating that gremlin-1 expression modulates the pulmonary inflammatory response to particulate exposure. Staining of silica treated wild type lung tissue with CD4 and CD8 T-cell markers as well as CD45R (B220) antibody, which recognizes mainly B-cells, indicated that both T- and B-lymphocytes were found in the aggregates (Fig 2D).

### Reduced interferon induced gene program in transgenic lungs

**Fig 3:**
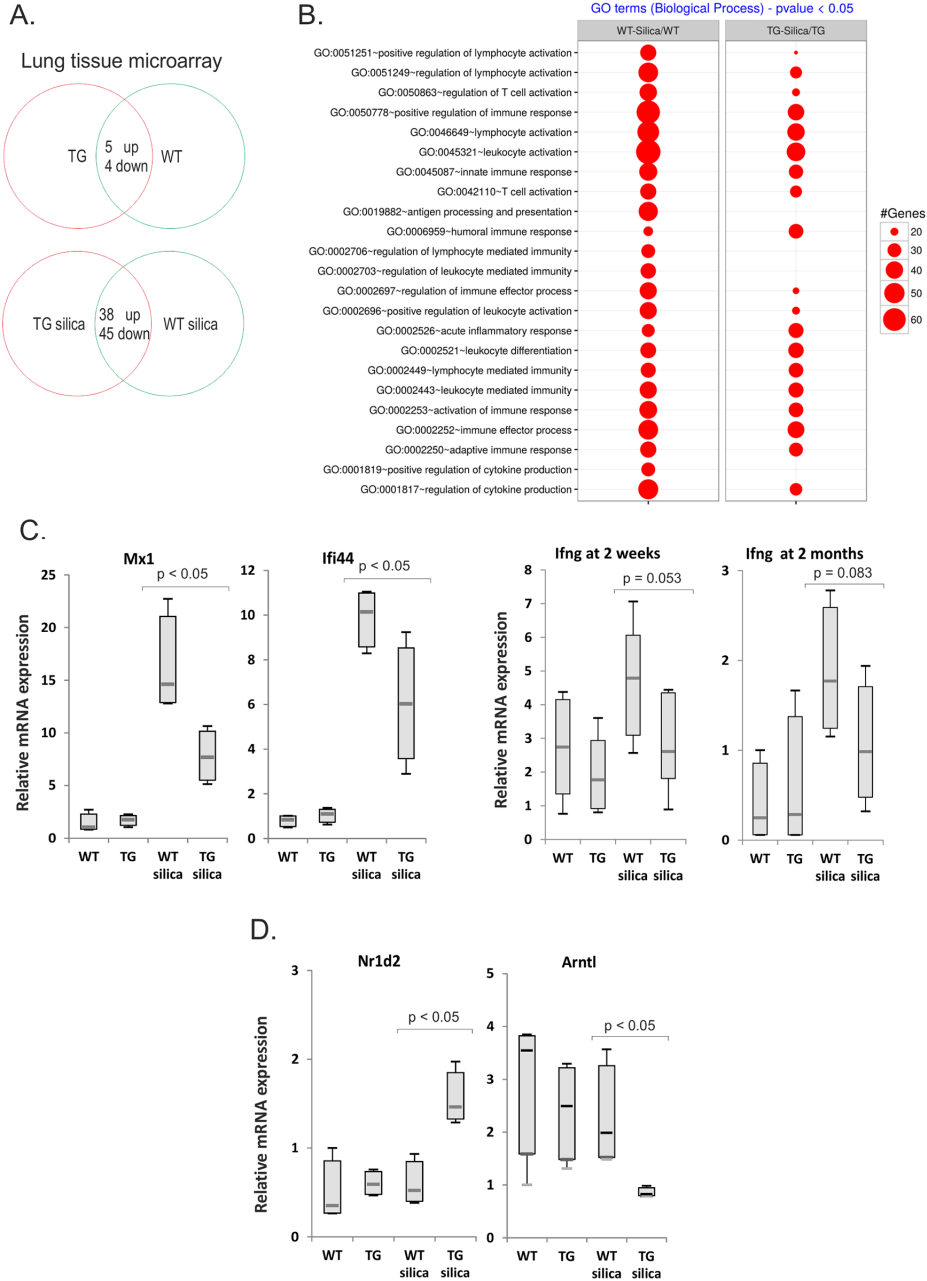
Reduced inflammatory gene response to silica. A. Gene expression microarray was performed using lung tissue mRNA isolated from 6 months old mice (n = 4 in each group). The number of upregulated or downregulated genes are indicated. B. Bubble plots for all immune-related annotations. It compares the most significant Gene Ontology (GO) terms from the “Immune-related Biological Process” ontology found across the different experimental conditions. The same selection strategy was applied for all conditions, which was a significance threshold of 0.05 for the adjusted enrichment p-value, at least five genes from the input list in the enriched category and the whole genome as reference background. C. and D. Quantitative RT-PCR analyses of selected genes identified as differentially expressed in the microarray. The results are presented as box blots. The p values were calculated using the Mann-Whitney U-test (at 2 weeks n = 8; at 2 months n = 4). WT = wild type mice; TG = gremlin-1 transgenic mice.

Microarray analysis was performed to characterize changes in gene expression in non-exposed or silica-exposed transgenic and wild type animals (see Methods). Gremlin-1 expression levels in lung tissue samples were determined by qPCR analyses since the microarray did not contain a probe that would recognize the transgene. *Grem1* mRNA levels were high in transgenic lungs as expected (S2B Fig). Only few genes were differentially expressed in transgenic lungs compared to wild type lungs, which is consistent with the minor histological findings (Fig 3A, Table 2 and S2C and S3 Figs). Silica exposure-induced robust changes in gene expression levels in both transgenic and wild type mice. The array results were visualized with a graphical BACA tool utilizing DAVID annotations [35]. Consistent with reduced lung inflammatory response, it was noted that immune response and immunity-related annotations were much less enriched in transgenic silica-exposed lungs (Fig 3B). Especially lymphocyte activation and cytokine production-related annotations were notably decreased. In addition, endogenous expression of *Grem1* was upregulated in both wild type and transgenic lungs, which is in agreement with our previous studies (S3 Fig) [8].

**Table 2:** Top genes differentially regulated in gremlin-1 transgenic lung.

Gene		logFC TG/WT	P.Value
**Angptl4**	Angiopoietinlike-4	1.08	0.0077
**Pdk4**	Pyruvate dehydrogenase kinase, isozyme 4	0.96	0.0042
**Upk3a**	Uroplakin 3A	0.81	0.0040
**Calcr**	Calcitonin receptor	0.77	0.0032
**Lamc3**	Laminin, gamma 3	0.64	0.0013
**Rab6b**	RAB6B, member ras oncogene family	-1.12	0.0027
**Chst8**	Carbohydrate (N-acetylgalactosamine 4–0) sulfotransferase 8	-0.82	0.0013
**Wif1**	WNT inhibitory factor 1	-0.76	0.0066
**MMP3**	Matrix metallopeptidase 3	-0.60	0.0010

Absolute Log2 fold change and p-values are shown for comparison of transgenic (TG) and wild type (WT) lungs.

Analyses of differentially expressed genes in silica-exposed mice indicated 38 upregulated and 45 downregulated genes in transgenic animals (>1.5-fold change, Table 3, S3 Fig). Most of the downregulated genes were linked to inflammatory responses (Fig 3B and 3C, Table 3). Quantitative RT-PCR verification was performed for selected genes (Fig 3C and S2B Fig). Especially interferon regulated genes, such as *Mx1*, *Rsad2*, *Ifi44* and *Stat2* were significantly less induced by silica exposure in transgenic mice. The expression of these genes also correlated with the amount of lymphocyte aggregates in the lung. In agreement, *Ifng* expression was significantly induced in wild type but not in transgenic silica-exposed mice (S3 Fig and Fig 3C). Direct comparison on silica-treated samples showed a clear trend of reduced *Ifng* expression (Fig 3C). Th1-cell surface marker *Cxcr3* was upregulated in wild type lung but not in transgenic lung following silica exposure, while a moderate induction of the Th2-cell surface marker *Ccr4* was observed in both (S3 Fig). These results are consistent with reduced Th1 inflammatory response in gremlin-1 transgenic lungs.

**Table 3:** Top genes differentially regulated in gremlin-1 transgenic lung.

Gene		logFC TG silic/WT silica	P.Value
**Dbp**	D site of albumin promoter binding protein	2.29	0.0007
**Chi3l4**	Chitinase 3-like 4	1.36	0.0007
**Nr1d2**	Nuclear receptor subfamily 1, group D, member 2	1.25	0.0007
**Gm6522**	Chitinase 3-like 3 pseudogene	1.22	0.0002
**Cyp2e1**	Cytochrome P450, family 2, subfamily E, polypeptide 1	1.15	0.0014
**Sftpb**	Surfactant protein B	1.00	0.0022
**Ces1g**	Carboxylesterase 1G	0.99	0.0021
**Cd207**	CD207 molecule, langerin	0.94	0.0032
**Tff2**	Trefoil factor 2	0.92	0.0048
**Calcr**	Calcitonin receptor	0.91	0.0009
**Lrat**	Lecithin retinol acyltransferase	0.87	0.0012
**Tspan4**	Tetraspanin 4	0.87	0.0069
**Ear11**	Eosinophil-associated, ribonuclease A family, member 11	0.86	0.0061
**Chi3l3**	Chitinase 3-like 3	0.85	0.0001
**Per3**	Period circadian clock 3	0.85	0.0048
**Klra2**	Killer cell lectin-like receptor, subfamily A, member 2	-1.19	0.0006
**Arntl**	Aryl hydrocarbon receptor nuclear translocator-like	-1.09	0.0034
**Oas2**	2'-5'-oligoadenylate synthetase 2, 69/71kDa	-1.07	0.0032
**Cr2**	Complement component (3d/Epstein Barr virus) receptor 2	-1.01	0.0087
**Rsad2**	Radical S-adenosyl methionine domain containing 2	-0.92	0.0017
**Nrcam**	Neuronal cell adhesion molecule	-0.89	0.0001
**Pydc3**	Pyrin domain containing 3	-0.88	0.0065
**Bst2**	Bone marrow stromal cell antigen 2	-0.86	0.0017
**Mx1**	Mx dynamin-like GTPase 1	-0.85	0.0017
**Isg15**	ISG15 ubiquitin-like modifier	-0.84	0.0039
**Ccnjl**	Cyclin J-like	-0.84	0.0005
**Gdpd2**	glycerophosphodiester phosphodiesterase domain 2	-0.83	0.0023
**Apol9b**	Apolipoprotein L 9b	-0.82	0.0098
**Klrd1**	Killer cell lectin-like receptor subfamily D, member 1	-0.79	0.0017
**Ifi44**	Interferon-induced protein 44	-0.77	0.0035

Absolute Log2 fold change and p-values are shown for comparison of silica-treated transgenic (TG) and wild type (WT) lungs.

Interestingly, several circadian clock transcription factors were differentially expressed in transgenic animals. Upregulation of negative regulators *Nr1d2* and *Per3* as well as downregulation of a central positive regulator *Arntl/Bmal1* was observed (Fig 3C and S2 Fig). In addition, *Dbp* was the most upregulated gene in transgenic silica-exposed lung. These findings link perturbations in circadian clock genes to gremlin-mediated inflammatory and tissue injury responses in the lung [38].

### Reduced chemokine levels in transgenic lungs after silica exposure for two weeks

To focus on the regulation of lung inflammatory responses by gremlin-1 expression, we performed a new *in vivo* experiment at an earlier time point, where inflammatory responses are expected to dominate. Two weeks after silica exposure, BAL fluid and lung tissue were collected for analysis of inflammatory cells and cytokines. Differential counts of BAL fluid cells indicated a tendency towards reduced percentage of lymphocytes in silica-treated transgenic mice (p = 0.108) (Table 4).

**Table 4:** Differential cell counts.

BAL cells %±SD	Monocytes /macrophages	Neutrophils	Lymphocytes	Atypical lymphocytes	Eosinophils
**WT**	95.2 ± 9.0	0.3 ± 0.5	4.5 ± 8.7	0	0
**TG**	97.9 ± 12.2	1.1 ± 0.8	0.8 ± 1.0	0	0.2 ± 0.4
**WT+silica**	50.6 ± 12.2	36.0 ± 14.4	**12.0 ± 7.5**	0.5 ± 1.4	0
**TG+silica**	42.6 ± 17.3	45.1 ± 18.7	**7.9 ± 4.3**	0.9 ± 1.2	0.1 ± 0.4

**Fig 4:**
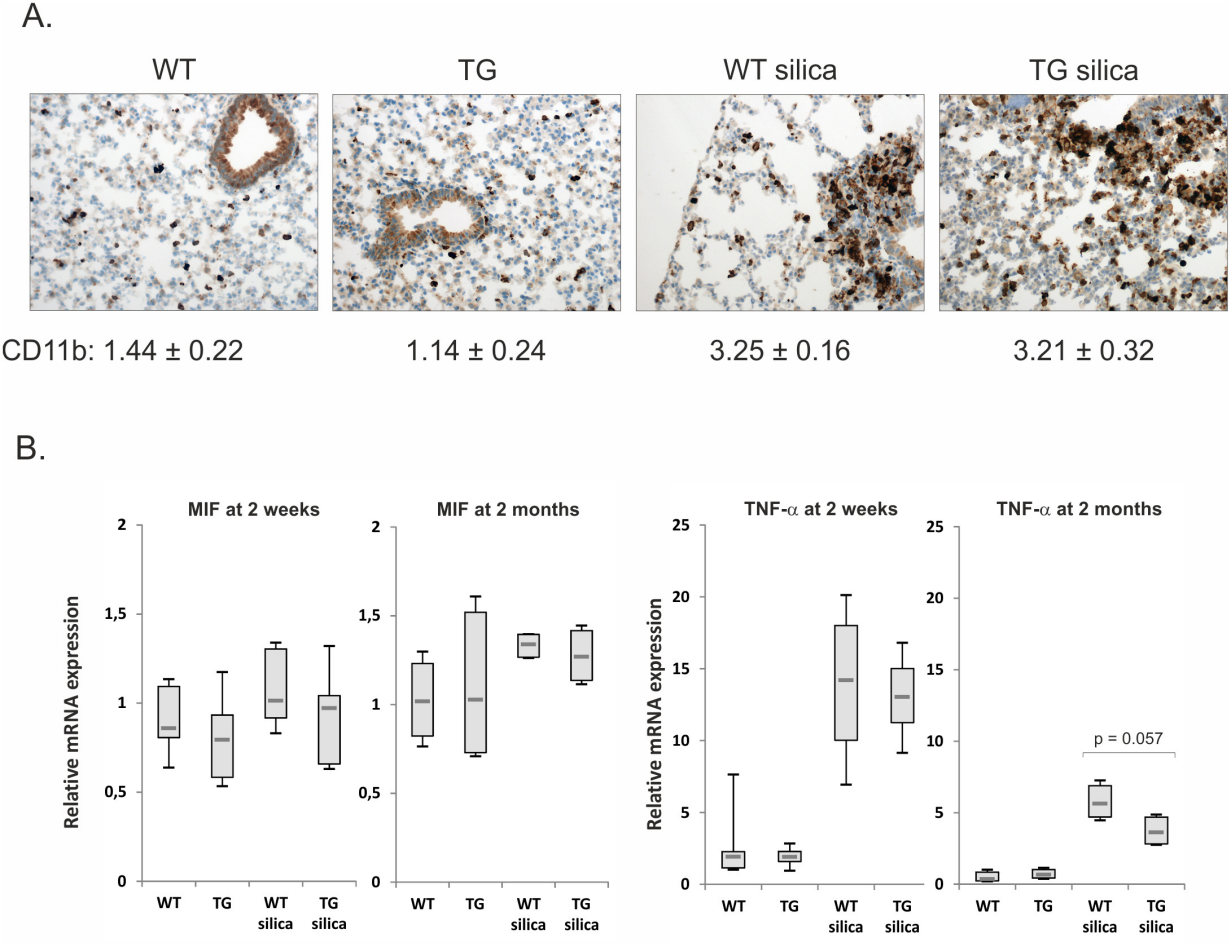
Gremlin-1 does not alter the overall innate immune response to silica. A. Immunohistochemical staining of lung tissue sections using CD11b antibody after two-week silica-exposure. Original magnification 200x. Staining scores are indicated below the photomicrographs (mean ± SEM, n = 8). B. Quantitative RT-PCR analyses of macrophage migration inhibitory factor (*Mif*) and tumor necrosis factor-α (*Tnf*) after two-week (n = 8) or two-month (n = 4) silica-exposure. The results are presented as box blots. The p values were calculated using the Mann-Whitney U-test. WT = wild type mice; TG = gremlin-1 transgenic mice.

Monocyte marker CD11b-staining was increased in silica treated lung tissue, but no difference was observed in the staining scores between wild type (3.25 ± 0.16 SEM) and transgenic (3.21 ± 0.32 SEM) lung (Fig 4A). CD11b is expressed in the surface of many leukocytes including monocytes, macrophages, natural killer cells and neutrophils, which suggests that gremlin-1 does not alter the overall innate immune response to silica. Gremlin-1 has been reported to inhibit MIF and macrophage differentiation as well as monocyte migration [22, 21]. Therefore, we analyzed the mRNA expression levels of *Mif* and *Tnf*, which is mainly produced by monocytes/macrophages in the lung. Differences in wild type versus transgenic expression were not observed (Fig 4B). These results indicate that gremlin-1 expression alters the lung inflammatory response by primarily reducing the recruitment of lymphocytes, instead of monocytes/macrophage-related mechanisms at the measured time point. Consistent with this, gene array results after two-month silica exposure indicated equal induction of *Cd68* and *Cd14* monocyte/macrophage markers in lung tissue (S3 Fig). At two months *Mif* expression was not altered in transgenic lungs, however, *Tnf* showed a trend towards decreased expression at this time point (Fig 4B).

**Fig 5:**
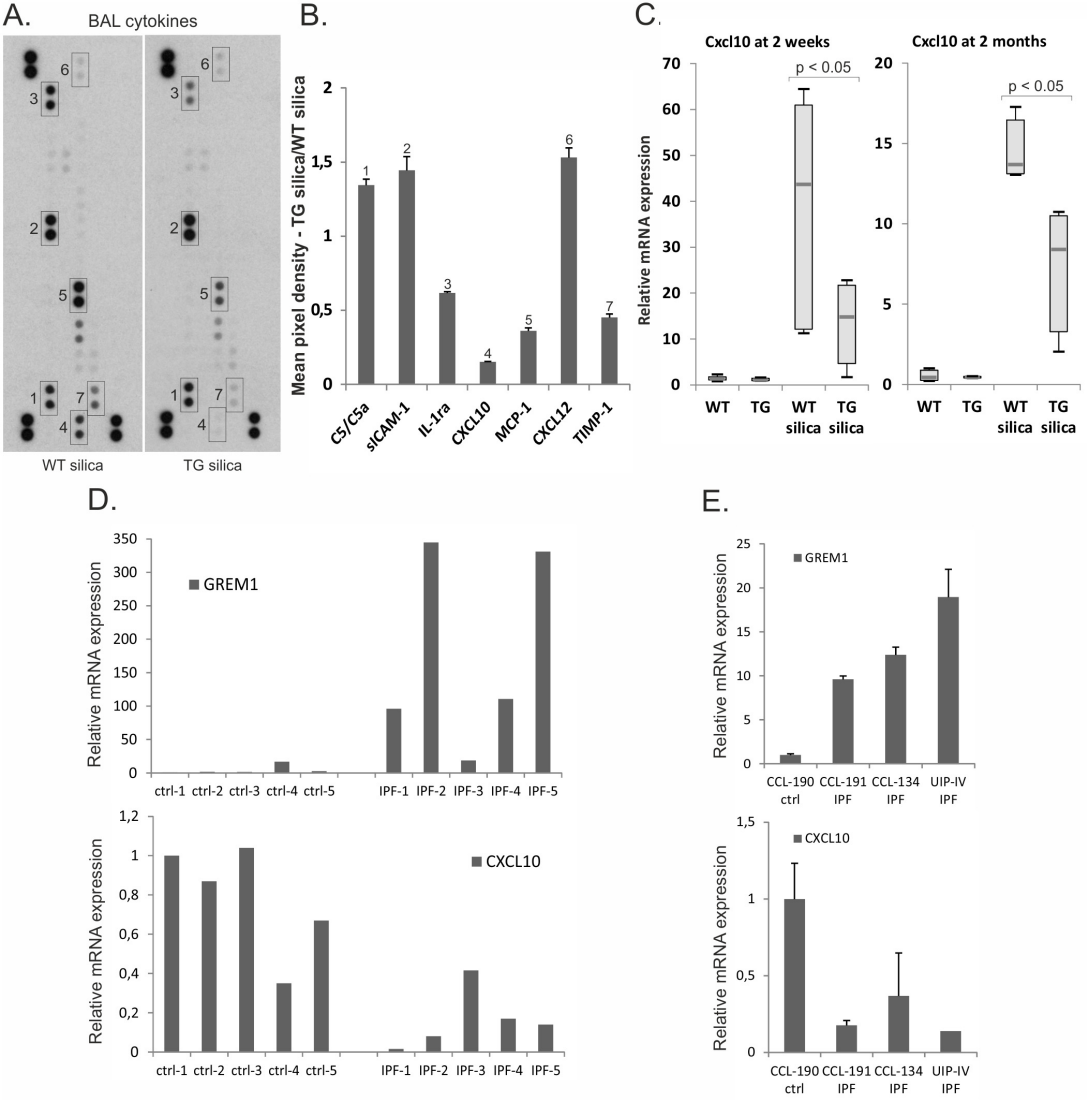
CXCL10 chemokine levels correlate negatively with gremlin-1 levels in mouse and human lung. A. Inflammatory cytokines in BAL fluid of wild type and gremlin-1 transgenic mice exposed to silica for two weeks were analyzed using a mouse cytokine array. B. Quantification of positive array signals. Mean pixel density of the signal in transgenic BAL fluid is divided by the signal in wild type BAL fluid. The error bars represent standard deviation (n = 2). C. Quantitative RT-PCR analyses of *Cxcl10* after two-week or two-month silica-exposure. The results are presented as box blots. The p value was calculated using the Mann-Whitney U-test (n = 8). WT = wild type mice; TG = gremlin-1 transgenic mice. D. Quantitative RT-PCR analyses of human gremlin-1 (*GREM1*) and *CXCL10* in control (ctrl) and idiopathic pulmonary fibrosis patient (IPF) lung tissue. E. Cultured human fibroblasts isolated from control (ctrl) and IPF patient lung tissue (IPF) were analyzed for *GREM1* and *CXCL10* expression by quantitative RT-PCR. The error bars represent standard deviation (n = 3).

The amount of inflammatory cytokines in BAL fluid was analyzed using a mouse cytokine array (see Methods). In the BAL fluid of wild type mice exposed to silica for two weeks the most abundant molecules were sICAM-1, MCP-1/CCL2, IL-1ra, C5/C5a, IP10/CXCL10 and TIMP-1 (Fig 5A and 5B). In transgenic BAL fluid the levels of several cytokines decreased, CXCL10 and CCL2 being the most downregulated cytokines. CXCL10 is an antifibrotic and angiostatic chemokine expressed by monocytes, endothelial and fibroblastic cells and an important T-lymphocyte chemoattractant [39]. Reduction in the amount of CXCL10, a Th1 cytokine, is in agreement with the observed reduction in lymphocyte recruitment. Furthermore, analysis of lung tissue mRNA expression levels indicated reduced *Cxcl10* expression both at 2 weeks and at 2 months after silica-exposure (Fig 5C). *Ccl2* mRNA expression in lung tissue was not significantly reduced (data not shown). CCL2 induces monocyte and macrophage migration, but has also anti-fibrotic effects in cultured human fibroblasts [40].

### Negative correlation between gremlin-1 and CXCL10 expression in IPF tissue and cultured fibroblasts

Decreased levels of CXCL10 have been previously associated with IPF [41, 42]. We analyzed mRNA expression levels of gremlin-1 and CXCL10 in control and IPF patient lung tissue samples. Gremlin-1 expression increased and CXCL10 expression decreased significantly in IPF lung tissue (Fig 5D and S4 Fig). A strong negative correlation was found in the expression levels of CXCL10 and gremlin-1 (r_s_ = -0.891, p = 0.001, n = 10). In addition, cultured fibroblasts isolated from IPF patients showed a similar negative association of mRNA expression levels (Fig 5E).

## Discussion

High gremlin expression has been functionally linked to malignant and fibrotic lung diseases. In IPF patients gremlin-1 expression levels are high and correlate with poor lung function [5, 6]. Experimental overexpression of gremlin-1 in mouse lung leads to severe developmental problems [4], which prevents studies on adult lung disease mechanisms. Transient overexpression of gremlin in rat lung results in epithelial activation and transient fibrotic changes, suggesting a role for gremlin-1 in promoting fibrosis [9]. We generated a transgenic mouse model to study the role of gremlin-1 in adult lung homeostasis and injury repair. Using the SPC promoter and a Cre-loxP system, gremlin-1 expression was specifically targeted to type II lung epithelial cells. Gremlin-1 transgenic mice were viable and showed no signs of respiratory insufficiency, indicating that epithelial gremlin-1 expression does not alter adult lung physiology. In addition, gene array analysis indicated only few alterations in lung gene expression levels in transgenic mice. Moderate pleural thickening and possible alveolar space enlargement detected at 6 month old animals were presumably caused by a minor interference with postnatal lung development.

Silica-exposed mice develop a neutrophilic inflammatory response and a progressive patchy lung fibrosis, which has similar features to human IPF. Gremlin-1 expression is induced in both asbestos- and silica-induced pulmonary fibrosis models [7, 8]. Transgenic overexpression of gremlin-1 in type II epithelial cells did not induce fibrosis or potentiate silica-induced fibrosis when analyzed after two months exposure. We have previously shown that gremlin-1 expression is functionally linked to the progression of fibrosis induced by asbestos-exposure in mouse lung [7]. In this model, gremlin-1 expression is induced and becomes apparent at day three after exposure and is significantly increased at two weeks [7]. In our transgenic model, we did not see any increase in fibrotic response, although in a rat model such effects have been shown using gremlin overexpression alone [9]. It is possible that the observed decreased lymphocytic response may regulate the following fibrotic response, although it has been suggested that innate immune processes are sufficient for the initiation of silica-induced fibrosis in mice [37]. There is also a possibility that the effects of gremlin-1 on post-natal lung development may be reflected in the adult injury response and development of fibrosis.

Similar to what we observed in the lung, transgenic gremlin-1 expression in mouse kidney alone has not induce fibrotic alterations or other phenotypic changes [43]. However, proximal tubular cell expression of gremlin-1 has been found to aggravate kidney fibrosis induced by streptozotocin [44]. In that particular model tagged human gremlin-1 protein was expressed in mouse proximal tubular epithelial cell. We expressed mouse gremlin-1 without any tags to ensure that the transgene functions as the endogenous protein and does not elicit non-specific responses. However, the results from kidney models suggest that gremlin-1 may act locally and in a cell and tissue-specific fashion. This is also suggested by the increased levels of pro-inflammatory factors observed following injection of recombinant gremlin-1 into the mouse kidney [24]. Similar to what has been found in endothelial cells [45], gremlin-1 was suggested to induce renal inflammatory responses through the activation of VEGFR2 in proximal tubular cells [24]. Anti-inflammatory functions of gremlin-1 have also been reported and include inhibition of monocyte migration and macrophage differentiation through BMP-independent mechanisms [21, 22]), again suggesting context dependent functions for gremlin-1.

The main new finding in this study was the specific decrease in silica-induced recruitment of lymphocytes into the gremlin-1 transgenic lung, while there was no obvious alterations in the overall innate immune response. Consistent with reduced number of lymphocyte aggregates in transgenic lungs, microarray results suggested a clear downregulation of the expression of inflammatory genes, particularly interferon response pathway genes. A number of genes, such as *Bst2*, *Rsad2*, *Ifi44*, *Oas2 and Stat2*, were previously found to be downregulated in pulmonary fibroblasts from IPF patients and from scleroderma-associated interstitial lung disease [46]. These results suggest local lung specific decrease in Th1 responses. Our current results indicate gremlin-1 as an important mediator of this shift in the balance of Th1/Th2 responses, which is a feature of IPF [47]. IPF and scleroderma patients lung tissue express high levels of gremlin-1 [5, 48]. A candidate gene for familial IPF, *ELMOD2*, has also been shown to regulate anti-viral responses, particularly interferon pathways suggesting a common mechanism [49]. The Th1 chemokine CXCL10 protein levels in the BAL fluid and lung tissue mRNA expression were found significantly reduced in transgenic silica-exposed mice. CXCL10 is an anti-fibrotic chemokine and has been strongly linked to the progression of fibrosis in mouse models. CXCL10 deficient mice exhibit increased pulmonary fibrosis after bleomycin treatment while overexpression of CXCL10 in mice reduces fibroblast accumulation and fibrosis suggesting that CXCL10 acts as a protective cytokine in the lung [50]. In gremlin-1 transgenic mice, however, clear alterations in the progression of fibrosis were not noted.

A number of studies have shown that CXCL10 and its receptor CXCR3 are involved in the regulation of inflammatory, angiogenic and fibrotic processes also in human lung diseases [42]. CXCL10 is involved in the selective recruitment of pro-inflammatory Th1-cells, which are characterized by CXCR3 expression. CXCL10 levels are reduced in IPF patient BAL fluid. In addition, CD4 positive T-cells in IPF patient BAL fluid have significantly lower CXCR3 expression [42]. CXCL10 is produced by leukocytes, epithelial, endothelial and fibroblastic cells. It can also inhibit fibroblast migration through CXCR3 receptor independent, syndecan-4 dependent manner, and this way act as an inhibitor of fibrotic processes [51]. We established a negative correlation in CXCL10 and gremlin-1 mRNA expression levels in control and IPF patient lung tissue as well in cultured human lung fibroblasts. Thus, increased gremlin-1 levels may lead to decreased local CXCL10 mRNA and protein levels in the lung, which contribute to lymphocyte recruitment and fibroblast migration.

Another interesting finding was regulation of circadian clock genes by gremlin-1 in silica-exposed lungs. Inflammatory responses are known to be affected by perturbations of the molecular clock function, which contributes to lung function in airway diseases. Decline in circadian function has been linked especially to depression of immune responses and exacerbations in asthma and COPD [38]. Interestingly, gremlin-2 and BMP-signaling was recently suggested to be regulated by circadian rhythms in the fibrous tissue of tendon [52]. Our findings strength the link between gremlin-1 and downregulation of inflammatory interferon responses during fibrotic processes. The function of gremlin-1 as a regulator of angiogenic processes mediated by VEGFR2 activation may also contribute to lung injury responses and fibrogenesis. Consistent with this, we noticed that gene array annotations related to blood vessel development and vasculature development were enriched in transgenic silica-treated lungs. Interestingly, histological analyses of one year old transgenic mice revealed aberrantly positioned blood vessels. Further studies are needed to characterize this phenomenon.

The view of the role of inflammation in IPF has gradually changed. Traditional anti-inflammatory treatments (steroids) are not effective in IPF, however, it has become clear that immune cells are present and may contribute to the fibrotic processes in the IPF lung [53]. A shift of inflammatory balance from lymphocytic to myelomonocytic lineage could even explain the lack of effect of immunosuppressive therapies in human IPF, where gremlin-1 expression is abundant [5]. Our results suggest that in addition to regulation of epithelial-mesenchymal crosstalk during tissue injury, gremlin-1 can potentially modulate inflammatory cell recruitment and chemokine production in the lung making gremlin-1 a potential target for therapeutic intervention.

## Supporting Information

S1 Fig Gremlin-1 protein expression is detected after birth in transgenic mice. Gremlin-1 protein expression was analyzed by immunofluorescence staining of frozen lung tissue sections prepared at postnatal days 0 (P0) and 1 (P1) as well at 6 weeks. Few very weakly positive cells could be detected at P0. At P1 gremlin-1 positive cells were clearly detectable, but the intensity was lower than in adult animals.
(TIF)

S2 Fig Quantitative RT-PCR analyses of selected genes identified as differentially expressed in the microarray. Expression of *Tnc* at 2 weeks (A) as well as *Grem1* (B) and *Agnptl4*, *Dbp*, *Stat2* and *Rsad2* (C) at 2 months are shown. The results are presented as box blots. The p values were calculated using the Mann-Whitney U-test (n = 4). WT = wild type mice; TG = gremlin-1 transgenic mice.
(TIF)

S3 Fig Tables of differentially expressed genes in pairwise comparisons from the microarray experiment. Differentially expressed genes with p-value < 0.01 and absolute log2 fold change > 0.58 are shown. The NCBI Entrez Gene IDs, gene info and gene symbols are shown. Additionally, for each significant gene, the average expression across the microarray samples, the t-test value, the nominal p-value, the adjusted p-value and the B value are reported.
(XLSX)

S4 Fig Quantitative RT-PCR analyses of gremlin-1 (*GREM1*) and *CXCL10* in control (ctrl) and idiopathic pulmonary fibrosis patient (IPF) lung tissue. The results are presented as box blots. The p values were calculated using the Mann-Whitney U-test.
(TIF)
