# Supplementary figures and images for: Gremlin-1 Overexpression in Mouse Lung Reduces Silica-Induced Lymphocyte Recruitment – A Link to Idiopathic Pulmonary Fibrosis through Negative Correlation with CXCL10 Chemokine
Source: PLoS One. 2016 Jul 18;11(7):e0159010. doi: 10.1371/journal.pone.0159010 (PMC4948891; doi:10.1371/journal.pone.0159010)

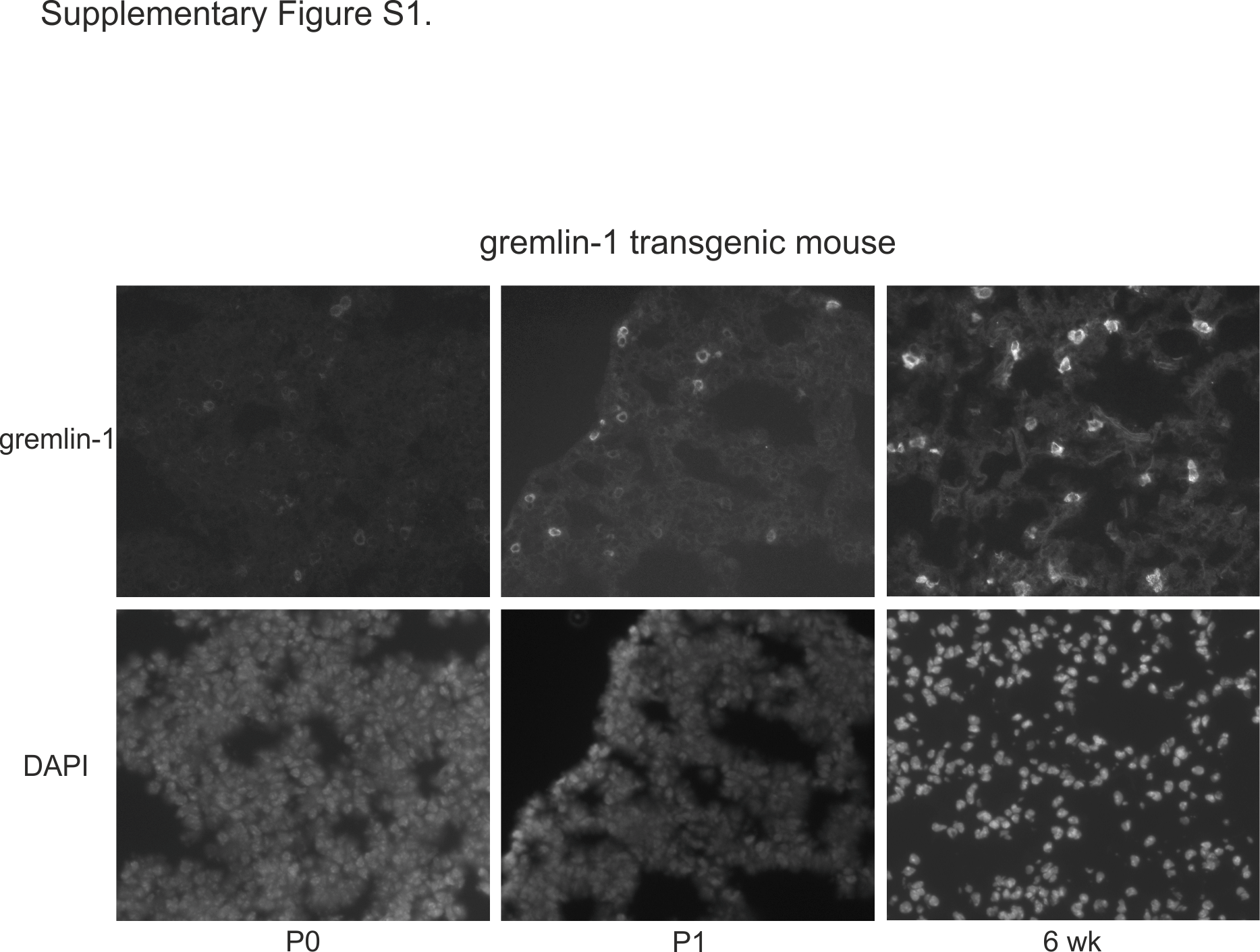

Supplement: S1 Fig — Gremlin-1 protein expression was analyzed by immunofluorescence staining of frozen lung tissue sections prepared at postnatal days 0 (P0) and 1 (P1) as well at 6 weeks. Few very weakly positive cells could be detected at P0. At P1 gremlin-1 positive cells were clearly detectable, but the intensity was lower than in adult animals. (TIF) [file pone.0159010.s001.tif]

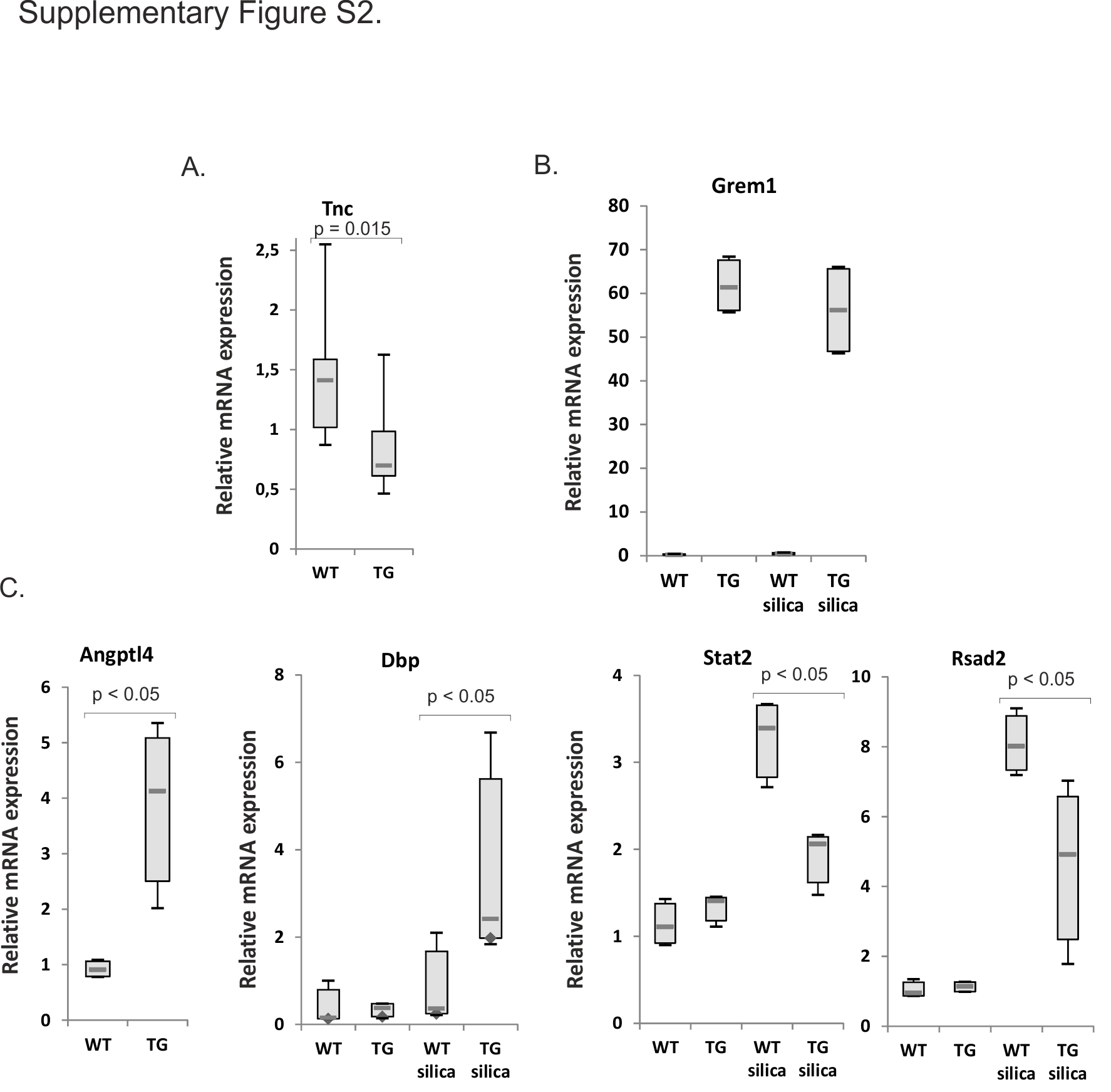

Supplement: S2 Fig — Expression of Tnc at 2 weeks (A) as well as Grem1 (B) and Agnptl4, Dbp, Stat2 and Rsad2 (C) at 2 months are shown. The results are presented as box blots. The p values were calculated using the Mann-Whitney U-test (n = 4). WT = wild type mice; TG = gremlin-1 transgenic mice. (TIF) [file pone.0159010.s002.tif]

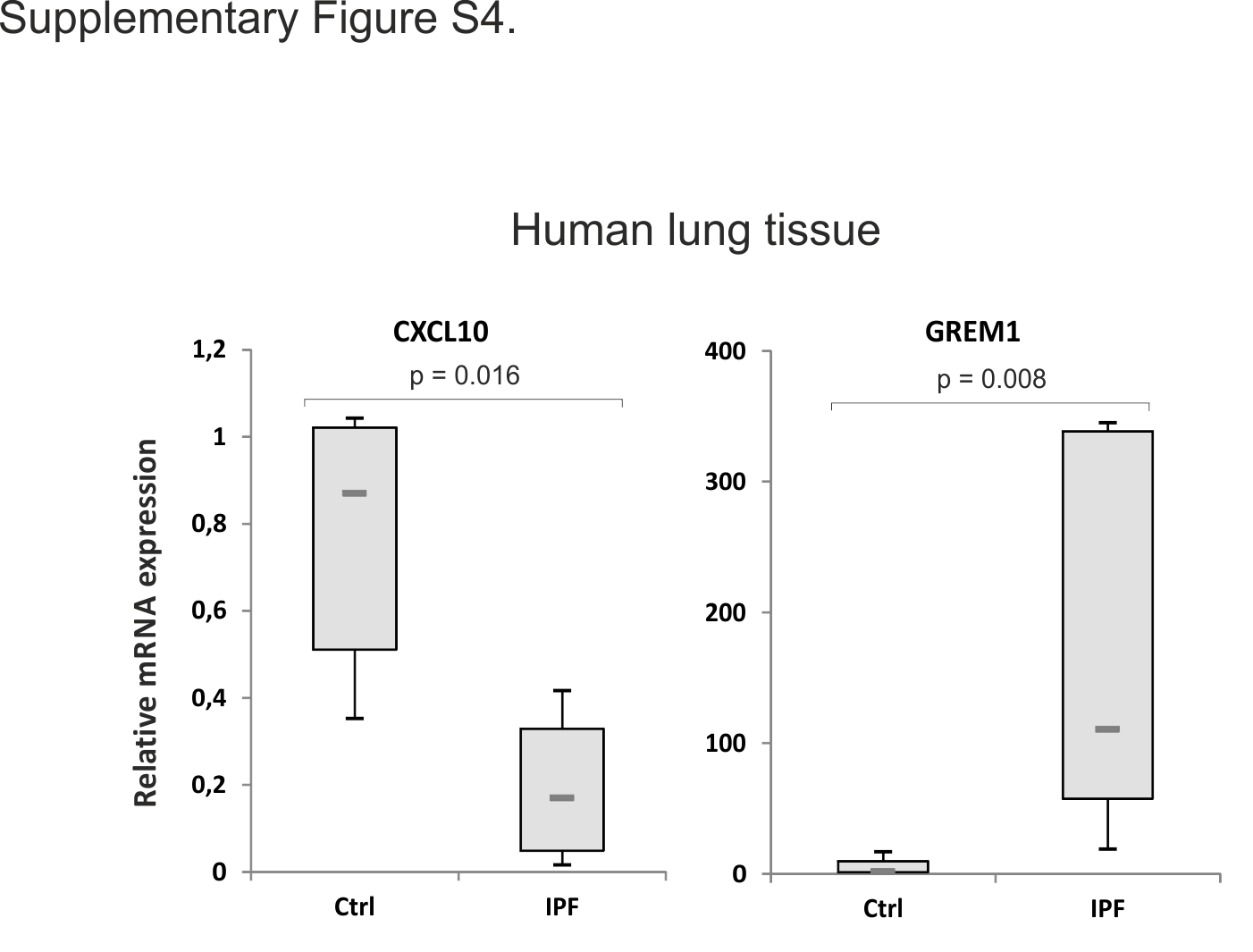

Supplement: S4 Fig — The results are presented as box blots. The p values were calculated using the Mann-Whitney U-test. (TIF) [file pone.0159010.s004.tif]
